# RNA Amplification Protocol Leads to Biased Polymerase Chain Reaction Results Especially for Low-Copy Transcripts of Human Bone Marrow-Derived Stromal Cells

**DOI:** 10.1371/journal.pone.0141070

**Published:** 2015-10-20

**Authors:** Carolin Coenen, Stefanie Liedtke, Gesine Kogler

**Affiliations:** Institute of Transplantation Diagnostics and Cell Therapeutics, Heinrich-Heine-University Medical Center, 40225, Düsseldorf, Germany; Charles P. Darby Children's Research Institute, 173 Ashley Avenue, Charleston, SC 29425, UNITED STATES

## Abstract

The amplification of RNA is becoming increasingly important, as often only limited amounts of cells are available for gene expression analysis. In this study, the gene expression profile of the 39 human homeobox (*HOX*) genes was analyzed in bone marrow-derived multipotent stromal cells (BM-MSCs) by reverse transcription (RT-) and quantitative polymerase chain reaction (qPCR). For further unlimited gene expression analysis, Whole Transcriptome Amplification (WTA) was used to amplify RNA from human BM-MSCs. However, WTA led to biased RT- and qPCR results, and even non-detectability of *HOX* transcripts compared to non-amplified BM-MSC samples which instead revealed transcription. It is important to note that the same RNA of the respective human BM-MSC line was used for normal cDNA synthesis by standard reverse transcription (non-amplified RT samples) and for cDNA synthesis by WTA (amplified WTA samples). On this account, the different RT- and qPCR results were unexpected applying WTA. The significantly reduced detection of *HOX* transcripts after WTA has been demonstrated for numerous BM-MSC lines (n = 26) by RT-PCR analysis. Furthermore, undetectable *HOX* transcripts meaning *HOX* transcripts of human BM-MSCs that were detected after normal cDNA synthesis, but were no longer detectable after WTA, were consistently observed by qPCR analysis. Finally, qPCR experiments revealed a possible explanation for the differences between amplified and non-amplified BM-MSC samples: an inverse correlation between the biased qPCR results and the low expression level of the respective *HOX* gene. The PCR analysis of high-copy transcripts like *GAPDH* or *RPL13A* revealed unchanged qPCR results after WTA compared to corresponding non-amplified BM-MSC samples. In contrast, WTA led to biased qPCR results for medium-copy *HOX* transcripts, and even non-detectability of low-copy *HOX* transcripts of human BM-MSCs resulting in false negative RT- and qPCR data applying WTA.

## Introduction

The analysis of transcriptome profiling provides understanding of important biological processes in cancer or other diseases. However, often only limited amounts of cells and correlating RNA are available for molecular biological analysis like cDNA microarrays [1], next generation sequencing [2] or polymerase chain reactions [3]. RNA amplification offers unlimited gene expression analysis of limited amounts of raw material. The challenge of RNA amplification is to represent the original transcriptome.

In this study, Whole Transcriptome Amplification (WTA) method developed by Qiagen [4] was used to amplify RNA from human bone marrow-derived multipotent stromal cells (BM-MSCs). The WTA method is modified from the Whole Genome Amplification (WGA) method based on Multiple Displacement Amplification (MDA) [5, 6]. The special feature of MDA compared to other PCR-based methods is that the DNA amplification proceeds at a constant temperature of 30°C. The DNA amplification without thermal cycling is based on the extraordinary processibility of the enzyme *phi29* polymerase synthesizing DNA fragments up to a length of 100 kb [7]. After *phi29* polymerase tightly binds to random primers, the enzyme replicates the DNA template without dissociating from the DNA template strand. Instead, *phi29* polymerase displaces complementary DNA strands and resolves secondary structures. The hybridization of further primers at the displaced DNA strands leads to additional replication resulting in DNA products larger than 10 kb. The long and branched DNA products synthesized by *phi29* polymerase ensure that MDA enables the representative amplification of human genomic DNA [5]. An advantage of *phi29* polymerase compared to thermostable *Taq* polymerase usually used in PCR-based methods, is their 3’− 5’ exonuclease proofreading activity resulting in significantly lower error rate of 1 in 10^6^−10^7^ [8] in contrast to normal *Taq* polymerase with an error rate of 3 in 10^4^ [9].

However, MDA cannot be used for the amplification of RNA or more precisely of transcribed cDNA, because the enzyme *phi29* polymerase only recognizes cDNA fragments longer than 2 kb. Therefore, a modified MDA approach for transcriptomic analysis has been developed by Qiagen [4]. To amplify also cDNA fragments smaller than 2 kb, a ligation step was added prior to MDA. In principle, the WTA method consists of three steps: 1. reverse transcription to generate cDNA, 2. ligation of short cDNA fragments to obtain template cDNA longer than 2 kb, 3. amplification of ligated cDNA by *phi29* polymerase.

The *HOX* genes belong to the family of homeodomain-containing transcription factors acting as master transcriptional regulators [10]. During embryonic development, *HOX* genes determine the positional identity along the anterior-posterior body axis [11]. However, *HOX* genes are not only expressed during early development of vertebrates, but also in normal adult cells indicating tissue specific expression [12–14]. In human, 39 *HOX* genes are distributed among four *HOX*-clusters (A-D) on four different chromosomes (7p15, 17p21, 12q13 and 2q31). The term *HOX*-code describes the specific expression of functional active *HOX* genes. Our research group revealed the *HOX*-code as a “biological fingerprint” to distinguish functionally distinct stem cell populations derived from cord blood [15] and showed the adaption of native *HOX*-negative unrestricted somatic stromal cells (USSCs) to a positive *HOX*-code after co-culturing with *HOX*-positive cells [16].

To analyze whether the original transcriptome is still represented after WTA, the same RNA of the respective human BM-MSC line was applied for normal cDNA synthesis by standard reverse transcription (non-amplified RT samples) and for cDNA synthesis by WTA (amplified WTA samples). As readout, the gene expression profile of the 39 human homeobox (*HOX*) genes was analyzed for amplified and non-amplified human BM-MSC samples by RT- and qPCR analysis.

## Material and Methods

### Generation and Expansion of BM-MSCs

The human bone marrow-derived multipotent stromal cell (BM-MSC) lines were isolated using BM aspirated from the iliac crest of adult donors. The participants provide their written content. The ethical approval to isolate the stromal cells was obtained from the ethical review board of the Medical Faculty, University of Duesseldorf (study 3484). Cells were cultivated in medium consisting of DMEM, 30% fetal bovine serum (Invitrogen) and 1% Penicillin/Streptomycin/L-Glutamine (Lonza) under the standard conditions at 37°C, 21% O_2_ and 5% CO_2_ until reaching 80% confluence. The stromal cells were detached with 0.2% trypsin/EDTA solution.

To evaluate the cumulative population doublings (CPD), the total number of cells was calculated applying Eq 1:

$${\text{PD}}_{\text{x}}\;=\frac{\text{log}(\frac{{\text{N}}_{1}}{{\text{N}}_{0}})}{\text{log}(2)};\;\text{CPD}\;=\;\sum {\text{PD}}_{\text{x}}$$ (1)

N_1_: number of plated cells

N_0_: number of harvested cells

### Total RNA Extraction

Total RNA was extracted from cell samples at passage 3 or 4, applying the RNeasy Mini Kit (Qiagen) according to manufacturer’s instructions. The isolation was performed with the optional use of QIAshredder columns to homogenize the cell-lysate and RNase-free DNase I to digest remaining DNA. The RNA concentration and purity was measured using a Nanodrop ND-100 device (Thermo Fisher Scientific). The ratio of 260/280 nm and 260/230 nm was in a confident range of 2.

### Quality of RNA

The quality of RNA was examined by Agilent 2100 Bioanalyzer at the Genomics and Transcriptomics Laboratory of Biological Medical Research Center Düsseldorf allowing the calculation of RNA integrity number (RIN). RIN values can range from ten to one. A RIN value of ten represents intact RNA, while complete degraded RNA has a RIN value of one. In this study, only RNA samples with RIN values ≥ 9.8 were used representing very high RNA quality.

### Reverse Transcription

Reverse transcription was performed for 60 min at 50°C using the First-strand cDNA synthesis Kit (Invitrogen) and the enclosed oligo(dT)_20_ primer. Up to 1000 ng total RNA were converted into first-strand cDNA in a 20 μl reaction. All control reactions provided with this system were carried out to monitor the efficiency of cDNA-synthesis. Prior to PCR, the completed first-strand reaction was heat-inactivated at 85°C for at least 5 min. Finally, cDNA was treated with RNaseH according to the manufacturer´s protocol.

### Whole Transcriptome Amplification

For unlimited gene expression analysis, the QuantiTect Whole Transcriptome Kit (Qiagen) was used according to manufacturer’s instructions. This method synthesizes cDNA from total RNA by Whole Transcriptome Amplification and comprises three steps: reverse transcription, cDNA ligation and amplification. About 10 up to 100 ng of total RNA were added to RT mix. Furthermore, the high-yield reaction consisting of an 8 h amplification step was performed to receive high cDNA yield. Corresponding to the QuantiTect Whole Transcriptome Handbook, the amplified cDNA was diluted 1:250 with water, and directly used for RT- and qPCR analysis.

### RT-PCR

Reverse transcription (RT)-PCR was carried out with gene specific and mainly intron-spanning primers (Thermo Scientific). The primer sequences (S1 Table) were carefully examined and checked for their specificity by applying BLASTn. *GAPDH* was used as reference gene. Approximately, 50 ng of cDNA or 1 μl of 1:250 diluted amplified cDNA were used for subsequent RT-PCR analysis in a total volume of 25 μl containing 1x PCR-buffer, 0.2 μM of each primer, 1.5 mM MgCl_2_, 0.2 mM of each dNTP and 1 U *Taq* DNA Polymerase (Invitrogen) at the following conditions: (1) 2 min at 95°C for initial denaturation, (2) 30 sec at 95°C, 30 sec at 56°C, 30 sec at 72°C for 35 cycles, (3) 5 min at 72°C for final extension of PCR products. As negative control, RT-PCR was performed without cDNA template. PCR was performed on a Mastercycler ep gradient S (Eppendorf). Subsequently, 20 μl aliquots of the RT-PCR products and related controls were analyzed by electrophoresis on a 2% agarose gel.

### Quantitative PCR

Quantitative PCR analysis was performed with the suitable primers already applied for RT-PCR analysis (S1 Table). *GAPDH* was used as reference gene for normalization in all experiments. Quantitative PCR was carried out with SYBR® Green PCR Mastermix (Applied Biosystems) using 10 ng cDNA or 2.5 μl of amplified cDNA. All reactions were run in technical triplicates, on a Step One Plus device (Applied Biosystems). PCR reactions were performed in a total volume of 25 μl containing 12.5 μl SYBR® Green PCR Mastermix, 6.0 μl dH_2_O, 2.5 μl cDNA template and 2 μl (0.2 μM) of each primer at the following condition: (1) 10 min at 95°C, (2) 15 sec at 95°C and 1 min at 60°C for 40 cycles. Specificity of the PCR product was confirmed by analyzing the melting curve, respectively. The Step One Software v2.3 was used to run and analyze the comparative Ct experiments. The threshold was kept at 0.2 for all experiments. Eq 2 was applied to evaluate the relative gene expression:

$$\Delta \text{Ct}=({\text{Ct}}_{\text{gene}}\;-\;{\text{Ct}}_{\text{GAPDH}})$$ (2)

Differential gene expression was calculated by 2^-ΔCt^. Quantitative PCR results were documented as mean values of technical triplicates and biological replicates with standard error of the mean (SEM).

### Statistical Analysis

The data are presented as arithmetic means with a standard error of mean (SEM) of at least three independent experiments. Two-tailed unpaired t-tests were conducted with GraphPad Prism (v. 5.01) to determine significance. A Welch’s correction was applied in case of significantly different variances. P values lower than 0.05 were considered as significant (*, p = 0.01–0.05; **, p = 0.001–0.01; ***, p < 0.001).

## Results

### Expression Level of *HOX* Genes

**Fig 1:**
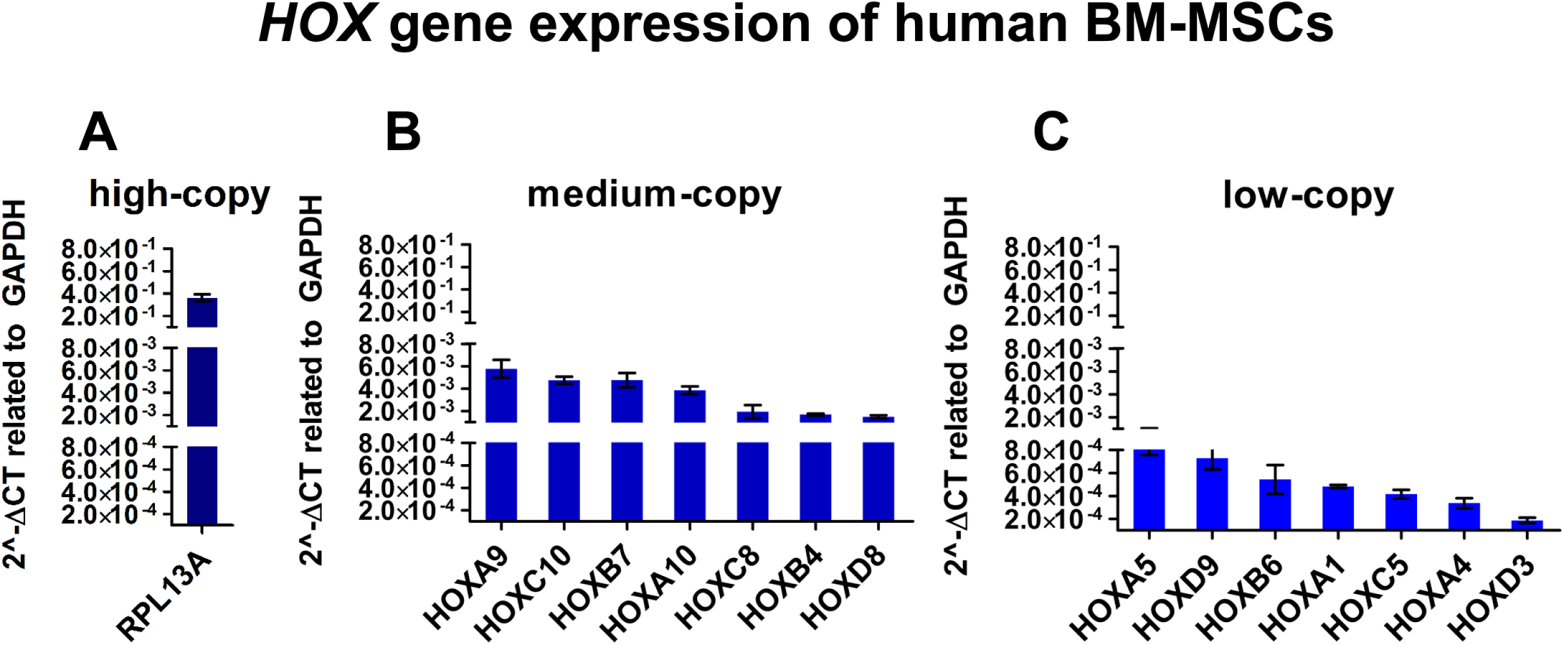
Expression level of *HOX* genes in human bone marrow-derived multipotent stromal cells (BM-MSCs). The expression level of seven medium-, and low copy *HOX* transcripts is documented for biological and technical replicates of BM-MSC lines (n = 3) by 2^-ΔCT^ value related to *GAPDH*. (A) *HOX* genes are weakly expressed in human BM-MSCs compared to high-copy transcripts like *RPL13A* showing an expression level of about 10^−1^ related to *GAPDH*. (B) The medium-copy *HOX* transcripts present an expression level of about 10^−3^ related to *GAPDH*. (C) The expression level of low-copy *HOX* transcripts decreases to 10^−4^ related to *GAPDH*.

The analysis of qPCR revealed that the expression level of *HOX* genes comprise a wide range in human BM-MSCs (Fig 1). Therefore, *HOX* genes are appropriate candidates to test WTA for unlimited PCR analysis of human BM-MSCs. In principle, *HOX* genes are weakly expressed in BM-MSCs compared to high-copy transcripts like *RPL13A* (Fig 1A) or *GAPDH* (data not shown). The high-copy *RPL13A* transcript revealed a threshold cycle (Ct) value of about 16. On basis of their expression level, *HOX* genes were graduated in groups of medium -, and low-copy transcripts. *HOX* genes with a Ct value between 22 and 25 were classified into the group of medium-copy transcripts. For example *HOXA9*, *HOXC10*, *HOXB7*, *HOXA10*, *HOXC8*, *HOXB4* and *HOXD8* represent medium-copy transcripts (Fig 1B). *HOX* genes revealing a Ct value between 26 and 29 were associated as low-copy transcripts. For instance, low-copy transcripts are *HOXA5*, *HOXD9*, *HOXB6*, *HOXA1*, *HOXC5*, *HOXA4*, and *HOXD3* (Fig 1C).

### Non-Detectability of *HOX* Transcripts after WTA

**Fig 2:**
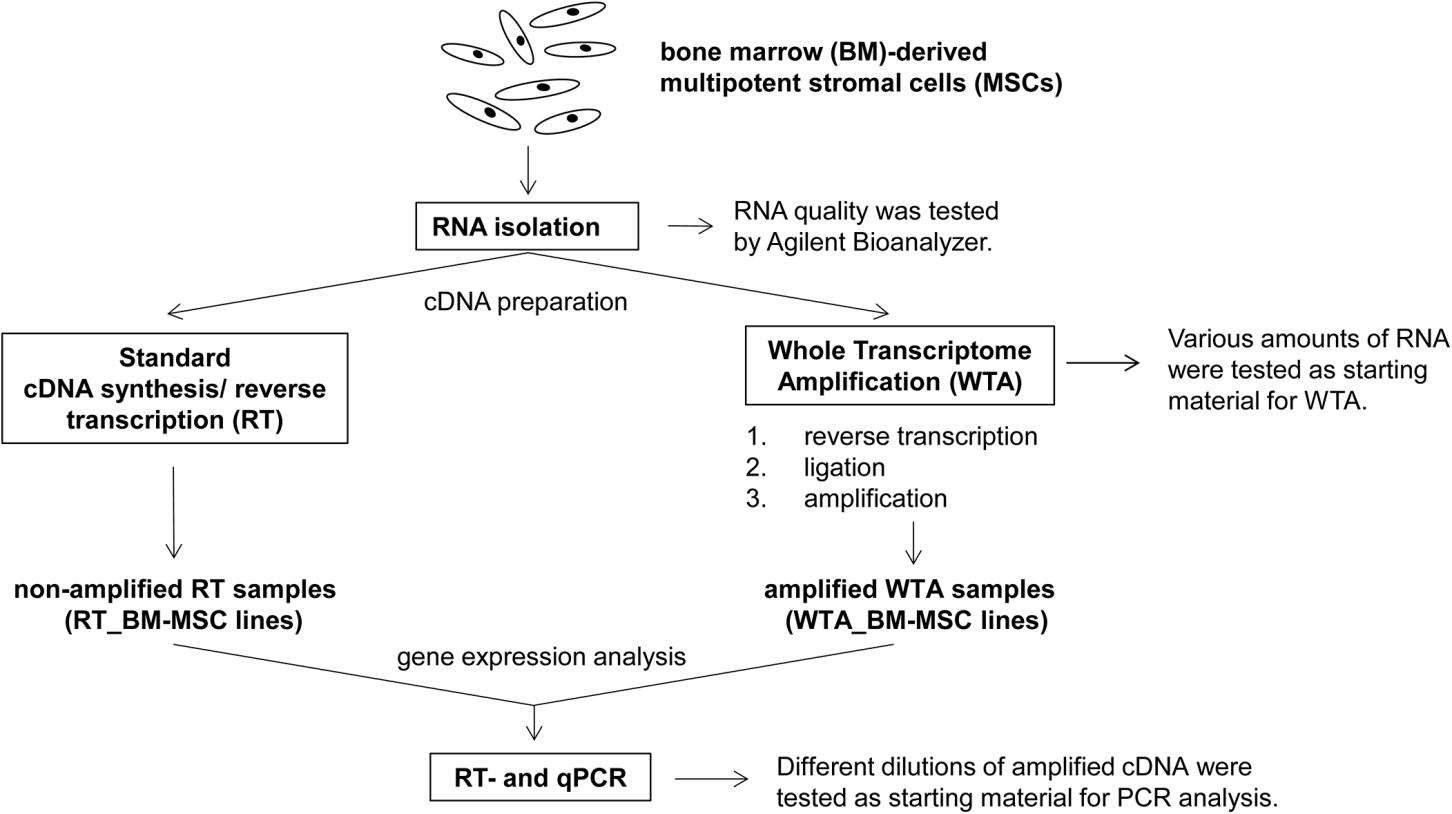
Overview of experimental structure. Human bone marrow-derived multipotent stromal cell (BM-MSC) lines were cultivated and expanded until passage 3 or 4. Following the RNA was isolated and the cDNA preparation was performed by standard reverse transcription and by Whole Transcriptome Amplification (WTA). The same RNA of the respective human BM-MSC line was applied for standard reverse transcription and WTA to compare RT- and qPCR results of the non-amplified and amplified cDNA samples. To exclude RNA degradation, the RNA integrity number (RIN) was determined by Agilent Bioanalyzer and only RNA samples with RIN values ≥ 9.8 were applied. Additionally, various amounts of RNA and cDNA were tested as starting material for WTA or for RT-PCR analysis.

The same RNA of the respective human BM-MSC line was used for normal cDNA synthesis by standard reverse transcription (non-amplified RT samples) or for cDNA synthesis by Whole Transcriptome Amplification (amplified WTA samples). Following RT- and qPCR analysis were performed and the PCR results of non-amplified and amplified BM-MSC samples were compared with each other (Fig 2).

**Fig 3:**
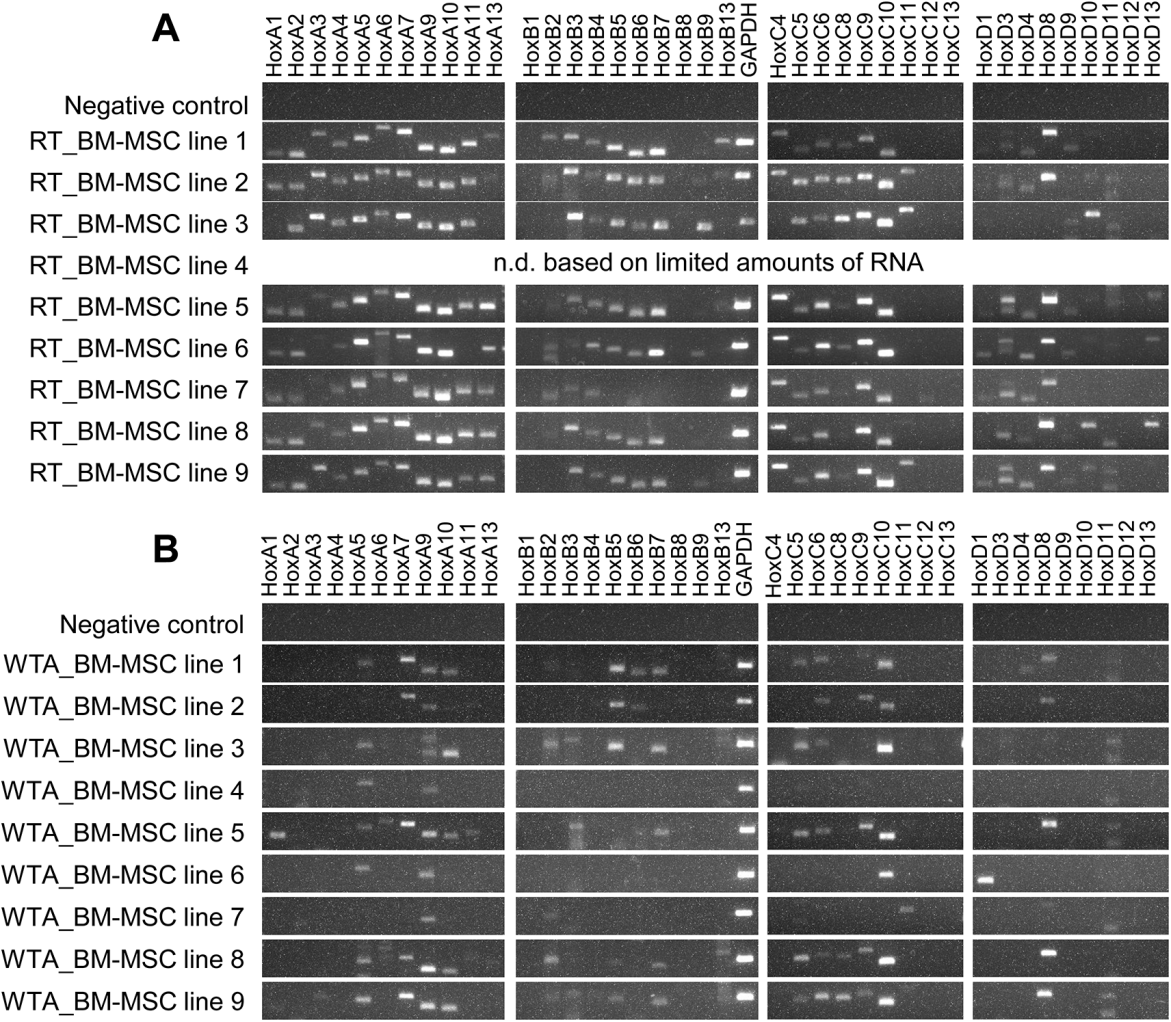
Non-detectability of *HOX* transcripts after Whole Transcriptome Amplification (WTA). In this experiment, the same RNA of the respective human bone marrow-derived multipotent stromal cell (BM-MSC) line (biological triplicates, n = 9) was applied for preparation of cDNA by standard reverse transcription (RT samples) and by Whole Transcriptome Amplification (WTA samples). (A) The so-called *HOX*-code of BM-MSCs is characterized by predominant gene expression of *HOXA*-, *HOXB*- and *HOXC*-cluster compared to *HOXD*-cluster. (B) After WTA, a significantly reduced detection of *HOX* transcripts was observed for all tested BM-MSC lines.

RT-PCR analysis of human BM-MSC lines revealed a characteristic *HOX* gene expression, the so-called *HOX*-code (Fig 3). The *HOX*-code is characterized by predominant expression of genes from the *HOXA*-, *HOXB*- and *HOXC*-cluster (Fig 3A) The majority of BM-MSC lines expressed the genes *HOXA1*, *HOXA2*, *HOXA4*, *HOXA5*, *HOXA6*, *HOXA7*, *HOXA9*, *HOXA10* and *HOXA11* from *HOXA*-cluster. The consistently expressed genes from *HOXB*-cluster were *HOXB4*, *HOXB5*, *HOXB6* and *HOXB7*. The constantly expressed genes from *HOXC*-cluster were *HOXC6*, *HOXC8*, *HOXC9* and *HOXC10*. The smallest number of *HOX* genes was expressed in *HOXD*-cluster. All BM-MSC lines revealed the expression of *HOXD8* and *HOXD9*, while the expression of *HOXD3* and *HOXD4* varied individually between the different BM-MSC lines. Generally, the BM-MSCs showed a reduced expression of the 5’-*HOX* genes from the *HOXB*-, *HOXC*- and *HOXD*-cluster. The majority of *HOX* genes, including the 5’-*HOX* genes, were expressed in the *HOXA*-cluster. The BM-MSCs expressed by mean 24 out of the 39 tested human *HOX* genes. But only about 10 *HOX* genes were still detectable after WTA (Fig 3B). It is important to note that the same RNA of the respective human BM-MSC line was used for normal cDNA synthesis by standard reverse transcription (non-amplified RT samples) and for cDNA synthesis by WTA (amplified WTA samples). On this account, the different RT-PCR results were unexpected applying WTA. The significantly reduced detection of *HOX* transcripts after WTA has been demonstrated for numerous BM-MSC lines (n = 26) by RT-PCR analysis. Evaluating the RT-PCR results revealed that mainly *HOX* genes like *HOXA5*, *HOXA7*, *HOXA9*, *HOXA10*, *HOXB5*, *HOXB7*, *HOXC6*, *HOXC10* and *HOXD8* were still detectable after WTA. The *HOX* transcripts of human BM-MSCs that were no more detectable after WTA are called undetectable *HOX* transcripts hereafter.

**Fig 4:**
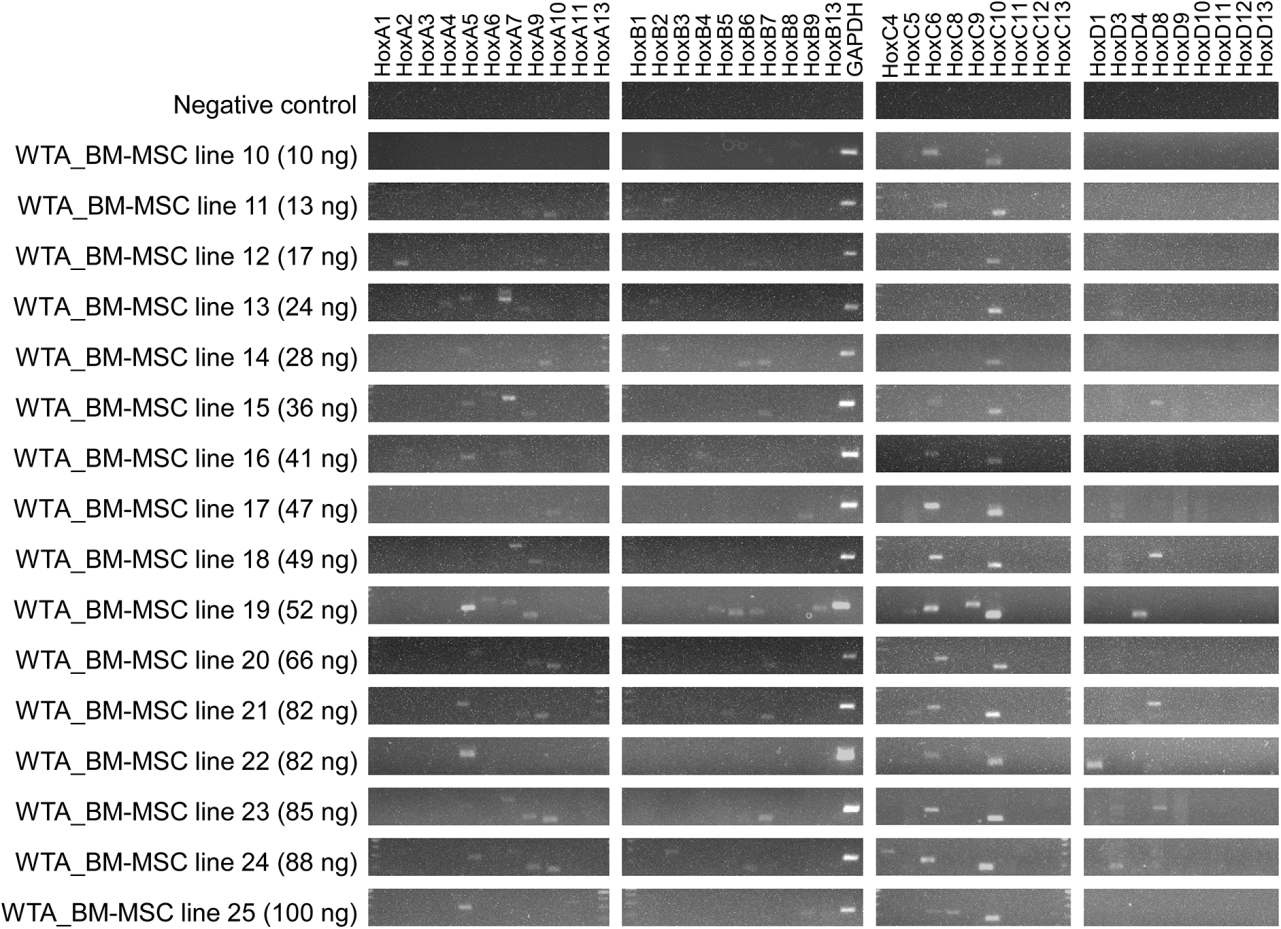
Various amounts of RNA as starting material for Whole Transcriptome Amplification (WTA). To exclude underrepresentation of low-copy *HOX* transcripts in the recommended amount of 10 ng RNA, between 10 ng up to 100 ng RNA were inserted to WTA reaction. RT-PCR analysis revealed consistently reduced detection of *HOX* transcripts for numerous BM-MSC lines (n = 16), despite gradually increasing amounts of RNA.

The RNA quality of human BM-MSC lines was tested to exclude RNA degradation. The RIN values ranged between 9.8 and 10.0 (data not shown) representing very high RNA quality. Additionally, various amounts of RNA as starting material for WTA were analyzed to test, if low-copy *HOX* transcripts are potentially underrepresented in the recommended amount of 10 ng RNA (Fig 4). Further on, RT-PCR analysis revealed significantly reduced detection of *HOX* transcripts after WTA for numerous BM-MSC lines (n = 16), even though gradually higher amounts of RNA template were used for WTA reaction.

**Fig 5:**
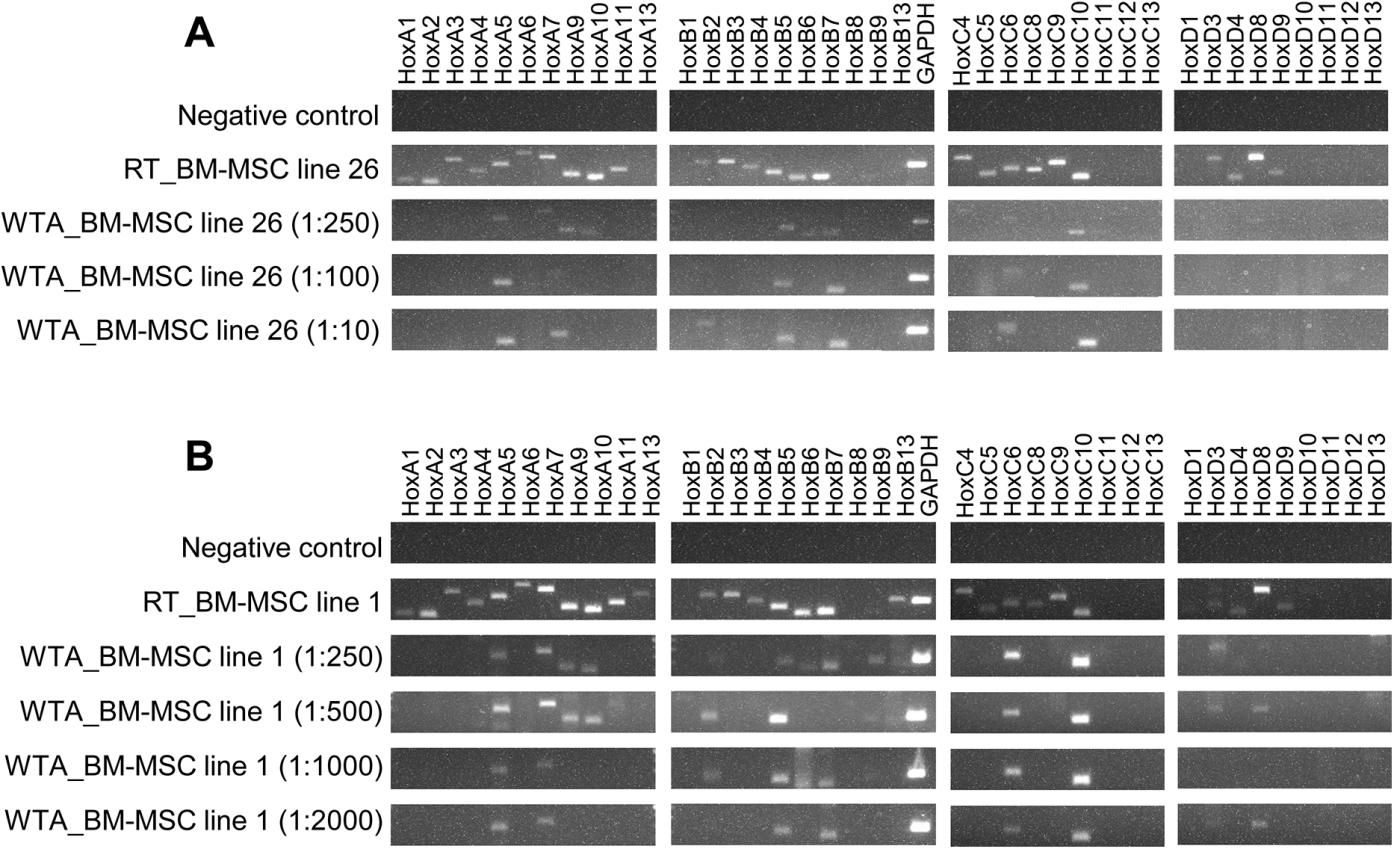
Different dilutions of amplified cDNA as starting material for RT-PCR analysis. The same cDNA from human bone marrow-derived multipotent stromal cell (BM-MSC) line 1 and 3 was constantly diluted for RT-PCR analysis. In brackets, the dilution of amplified cDNA is outlined after high-yield Whole Transcriptome Amplification (WTA) reaction (8 h amplification). (A) Lower cDNA dilutions (1:100, 1:10) led to more undetectable *HOX* transcripts like *HOXA9* or *HOXA10* compared to the recommended 1:250 cDNA dilution. (B) Higher cDNA dilutions (1:500, 1:1000, 1:2000) also revealed a higher amount of undetectable *HOX* transcripts like *HOXA9*, *HOXA10* or *HOXB9* compared to the recommended 1:250 cDNA dilution.

Furthermore, different dilutions of amplified cDNA as starting material for RT-PCR analysis were analyzed by dilution series (Fig 5). But lower (1:100, 1:10) and higher (1:500, 1:1000, 1:2000) cDNA dilutions led to a higher amount of undetectable *HOX* transcripts in RT-PCR analysis compared to the recommended 1:250 cDNA dilution. According to these results, the 1:250 cDNA dilution of the respective human BM-MSC line was used for further PCR analysis. In summary, neither the RNA quality nor the amount of RNA or cDNA seemed to be responsible for the undetectable *HOX* transcripts after WTA.

### Biased qPCR Results for Medium-Copy *HOX* Transcripts after WTA

**Fig 6:**
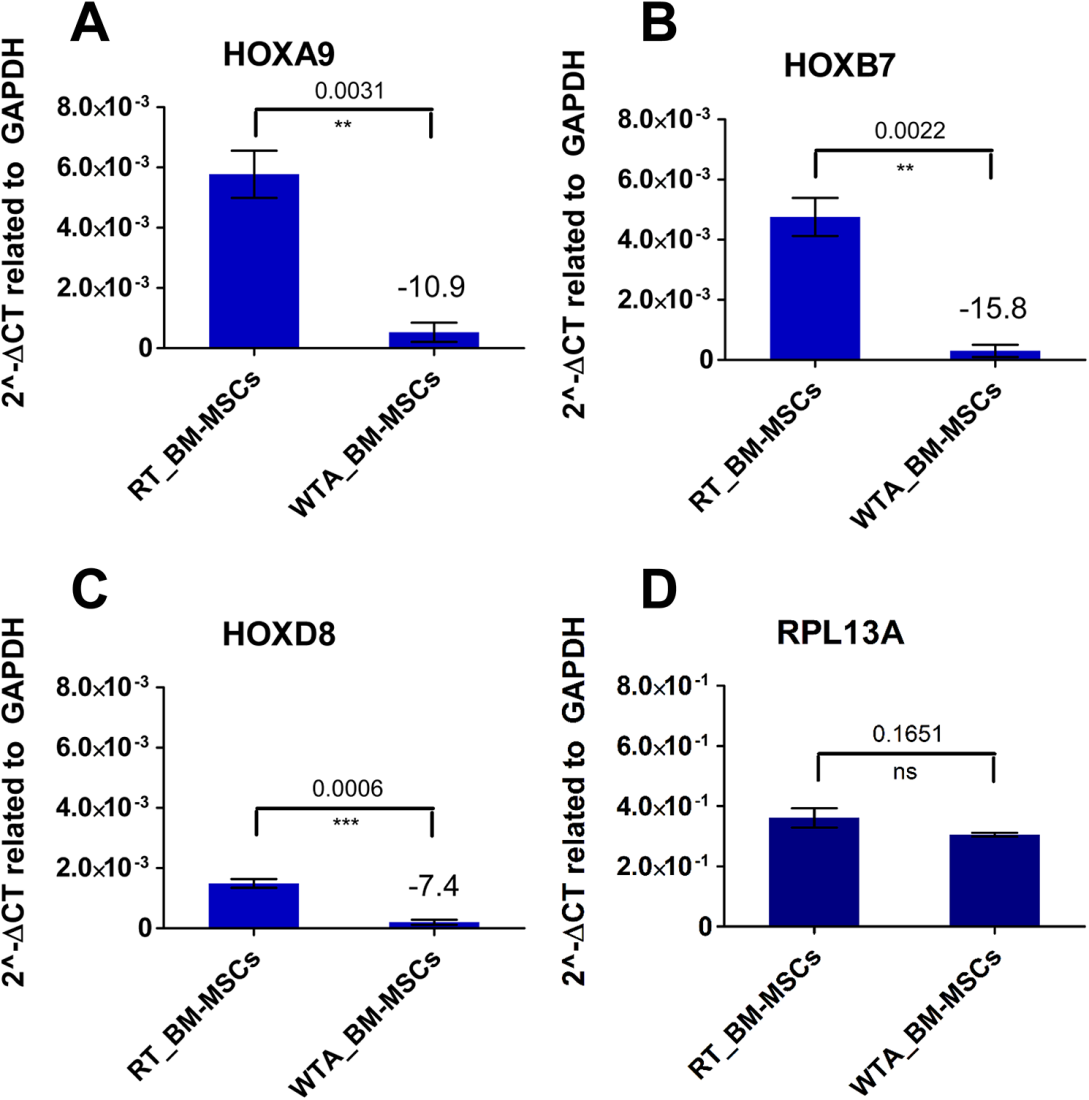
Biased qPCR results for medium-copy *HOX* transcripts after Whole Transcriptome Amplification (WTA). Similar to RT-PCR analysis, the same cDNA of the respective human bone marrow-derived multipotent stromal cell (BM-MSC) line (biological triplicates, n = 3) synthesized by standard reverse transcription (RT samples) and Whole Transcriptome Amplification (WTA samples) was used for qPCR analysis. (A-C) The WTA samples revealed a significantly decreased expression level of the medium-copy *HOX* genes like *HOXA9*, *HOXB7* and *HOXD8* in comparison to RT samples. (D) The reference gene *RPL13A* is expressed on a significantly higher level compared to the *HOX* genes resulting in no significantly different qPCR results after WTA.

Similar to RT-PCR analysis, the same cDNA of the respective human BM-MSC line synthesized by standard reverse transcription (non-amplified RT samples) and Whole Transcriptome Amplification (amplified WTA samples) was used for q PCR analysis. Exemplary, three medium- and low-copy *HOX* transcripts were examined in qPCR analysis. As a result, the amplified WTA samples of human BM-MSCs revealed biased expression level for medium-copy *HOX* transcripts in comparison to RT samples of the same BM-MSC lines (Fig 6). For example, the expression level of *HOXA9* reached a negative fold change of -10.9 after WTA (Fig 6A). Quantitative PCR analysis of *HOXB7* revealed also a significantly reduced expression level after WTA with a negative fold change of -15.8 compared to the same non-amplified human BM-MSC lines (Fig 6B). The expression level of *HOXD8* showed a negative fold change of -7.4 after WTA (Fig 6C). As control, the qPCR analysis was also performed with high-copy transcripts like *RPL13A* (Fig 6D) and *GAPDH* (data not shown). The expression level of *RPL13A* and *GAPDH* was not significantly different for human BM-MSCs with and without WTA.

### Non-Detectability of Low-Copy *HOX* Transcripts in qPCR Analysis after WTA

**Fig 7:**
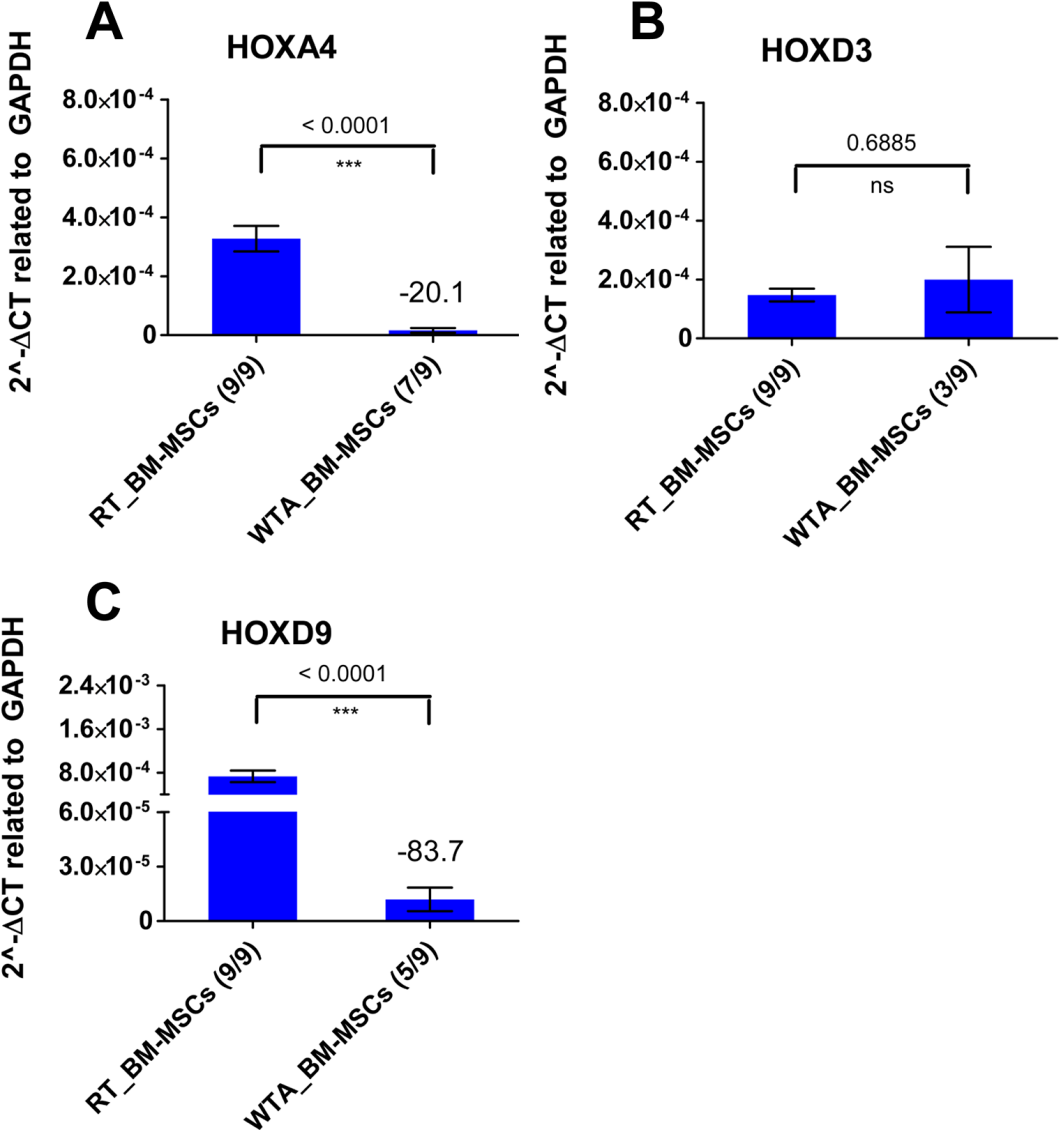
Non-detectability of low-copy *HOX* transcripts after Whole Transcriptome Amplification (WTA). Similar to RT-PCR analysis, the same cDNA of the respective human bone marrow-derived multipotent stromal cell (BM-MSC) line (biological replicates, n = 9) synthesized by standard reverse transcription (RT samples) or Whole Transcriptome Amplification (WTA samples) was used for qPCR analysis. (A-C) The number of samples out of the 9 tested samples is shown in brackets, where the respective low-copy *HOX* transcript could be detected in qPCR analysis. The low-copy *HOX* transcripts were undetectable in the remaining samples. In comparison to WTA samples, all RT samples of BM-MSC lines revealed *HOXA4*, *HOXD3* and *HOXD9* gene expression.

Quantitative PCR analysis of medium-copy *HOX* transcripts of amplified human BM-MSC lines (WTA samples) revealed on average a negative fold change of -11.4 in comparison to same non-amplified BM-MSC lines (RT samples). In contrast, qPCR analysis of the low-copy *HOXA4* and *HOXD9* transcripts of amplified human BM-MSC lines showed higher negative fold changes (Fig 7). The expression level of *HOXA4* reached a negative fold change of -20.1 after WTA. The expression level of *HOXD9* showed a negative fold change of -83.7 after amplification. In contrast, the expression level of the low-copy *HOXD3* transcript was not significantly different with and without WTA. However, a special feature was observed for low-copy *HOX* transcripts: The low-copy *HOXA4*, *HOXD3* and *HOXD9* transcripts could not always be detected in all tested WTA samples resulting in artificial qPCR data. For example, the expression of *HOXA4* transcript could only be detected in 7 out of 9 (7/9) tested WTA samples (Fig 7A). The low-copy *HOXD3* transcript was only established in 3/9 WTA samples (Fig 7B). The *HOXD9* expression was only detected in 5/9 WTA samples (Fig 7C). In comparison, the low-copy *HOXA4*, *HOXD3* and *HOXD9* transcripts of human BM-MSCs were detected in each of the 9 tested RT samples. Agarose electrophoresis was additionally performed with qPCR products to identify the specific PCR products, not only by their characteristic melt curves, but also by their characteristic product length (data not shown).

It can be concluded that the PCR analysis of high-copy transcripts revealed unchanged qPCR results for human BM-MSCs after WTA. In contrast, WTA led to biased qPCR results for medium-copy transcripts, and even non-detectability of low-copy transcripts of human BM-MSCs. Moreover, undetectable *HOX* transcripts of human BM-MSCs after WTA were consistently observed by RT- and qPCR analysis.

## Discussion

RNA amplification provides a tool to perform PCR analysis of limited amount of cells, and associated RNA. In this study, the gene expression profile of the 39 *HOX* genes was analyzed for human BM-MSCs by RT- and qPCR analysis. For further unlimited gene expression analysis, the isolated RNA was amplified by WTA method based on MDA.

One property of *HOX* genes is their wide range of different expression levels in human BM-MSCs. Furthermore, the majority of *HOX* genes only comprise one intron. Therefore, the *HOX* genes reveal similar structure and full-length transcripts between 800–5000 nucleotides, whereby the most *HOX* genes have transcript lengths of about 2000 nucleotides. As a result, the location of primers is selected very similar, nevertheless specific for each *HOX* gene. Their comparability and simultaneously their specificity and different expression levels in human BM-MSCs enable *HOX* genes as suitable candidates for the evaluation of WTA.

As result, WTA led to biased RT- and qPCR results, and even non-detectability of *HOX* transcripts compared to non-amplified BM-MSC samples. It is important to note that the same RNA of the respective BM-MSC line was used for normal cDNA synthesis by standard reverse transcription (non-amplified RT samples) and for cDNA synthesis by WTA (amplified WTA samples). On this account, the different RT- and qPCR results were unexpected applying WTA.

**Fig 8:**
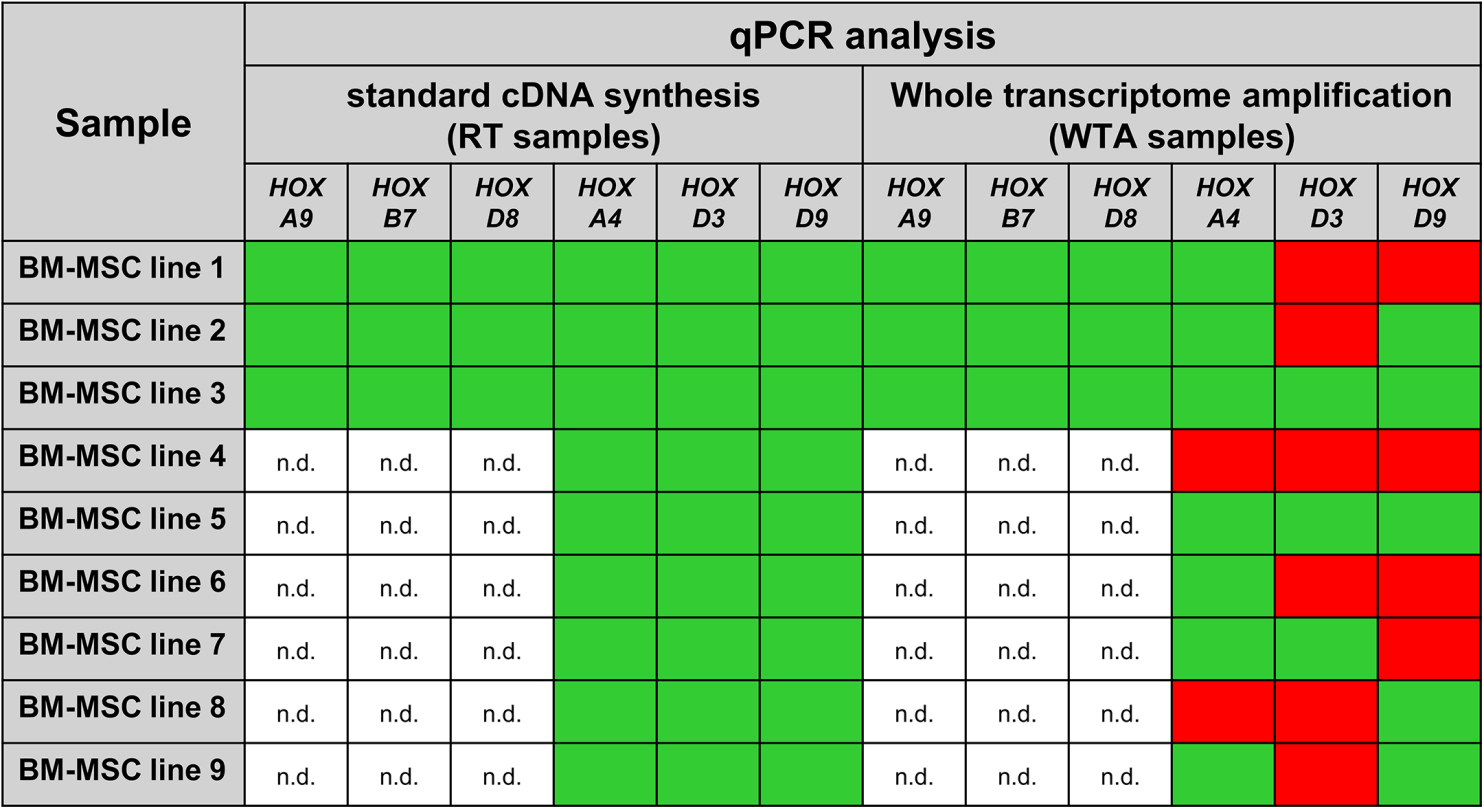
Summary of qPCR results for human bone marrow-derived multipotent stromal cells. When cDNA was synthesized by standard cDNA synthesis, the tested medium- and low-copy *HOX* transcripts could be detected by qPCR. In contrast, when cDNA was synthesized and amplified by WTA, the low-copy HOX transcripts were not fully converted to cDNA. n.d. = not determined.

RT-PCR results revealed significantly reduced detection of *HOX* transcripts after WTA for numerous BM-MSC lines (n = 26). It was conspicuous that mainly *HOX* genes like *HOXA5*, *HOXA7*, *HOXA9*, *HOXA10*, *HOXB5*, *HOXB7*, *HOXC6*, *HOXC10* and *HOXD8* were still detectable after amplification. Based on these results, it might be suggested that preferential more strongly expressed *HOX* genes, identifiable by strong DNA bands in RT-PCR gels, are still detectable after WTA. Accordingly, undetectable *HOX* genes meaning *HOX* transcripts of human BM-MSCs that were detected after normal cDNA synthesis, but were no more detectable after WTA, seemed to be more weakly expressed *HOX* genes. This assumption was confirmed by qPCR analysis (Fig 8). The medium-copy *HOX* transcripts *HOXA9*, *HOXB7* and *HOXD8* could be detected in all tested human BM-MSC lines (n = 9) after WTA by qPCR analysis. In contrast, the low-copy *HOX* transcripts *HOXA4*, *HOXD3* and *HOXD9* could only be detected in some of the BM-MSC lines after WTA, despite the expression of these low-copy *HOX* transcripts was established in the corresponding non-amplified BM-MSC samples by qPCR analysis. Correlating to qPCR analysis, the majority of medium-copy *HOX* transcripts could also be detected in RT-PCR analysis after WTA showing a DNA band in the RT-PCR gel of WTA samples. Likewise, the low-copy *HOX* transcripts *HOXA4*, *HOXD3* and *HOXD9* could not be detected after WTA by RT-PCR, despite the expression of these low-copy *HOX* genes was established in the corresponding non-amplified BM-MSC samples. Considering that qPCR is a more sensitive method compared to RT-PCR requiring a certain amount of DNA molecules to result in a visible DNA band on the RT-PCR gel, it is not surprising that some low-copy *HOX* transcripts could be detected by qPCR. Nevertheless, low-copy *HOX* transcripts got lost after WTA for human BM-MSCs. Moreover, qPCR analysis of high-, medium-, and low-copy transcripts revealed an intergradation reaching from not significantly different to biased and even undetectable *HOX* transcripts.

The represented qPCR data of human BM-MSCs can lead to the conclusion that the gene expression level may influence the efficiency of WTA. This assumption is confirmed by the observation that qPCR analysis of high-copy transcripts *RPL13A* and *GAPDH* revealed no significantly different PCR results after WTA. In contrast, WTA led to biased qPCR results for medium-copy *HOX* transcripts based on significantly reduced expression level of medium-copy *HOX* transcripts after WTA compared to the same non-amplified BM-MSC samples. Finally, WTA led to non-detectability of low-copy *HOX* transcripts for human BM-MSCs resulting in false negative RT- and qPCR data applying WTA.

Simultaneously, the identification of high- and medium-copy transcripts by RT- and qPCR analysis of human BM-MSCs after WTA gave evidence for functioning of WTA reaction and the correct technical application. But nevertheless, the significantly reduced expression level of medium-copy transcripts might be negligible, if only amplified cDNA samples are compared with each other. The analysis of up-regulated or overexpressed genes of amplified cDNA samples is also possible [17, 18]. However, the use of WTA is getting difficult, when the gene expression profile of amplified and non-amplified cDNA samples is analyzed and low-copy transcripts are no more detectable after WTA resulting in false negative RT- and qPCR data.

The quality and quantity of RNA as starting material for WTA reaction was tested to exclude that these factors limit the efficiency of WTA reaction. The Agilent analysis revealed high RNA quality of human BM-MSCs represented by RIN values between 9.8 and 10.0. To exclude underrepresentation of low-copy transcripts, larger amounts of RNA were inserted to WTA reaction. Despite higher starting amounts of RNA from 10 ng up to 100 ng, the reduced detection of *HOX* transcripts was consistently observed for human BM-MSCs indicating that not the absolute amount of RNA, but rather the expression level of high-, medium-, and low-copy transcripts seems to determine the efficiency of WTA reaction. Different cDNA dilutions were additionally tested to determine the suitable amount of cDNA template for RT-PCR reactions. As a result, the recommended 1:250 cDNA dilution was considered as adequate for PCR analysis. Another possible source of errors could be the unequal distribution of low-copy transcripts when RNA isolation is performed with less than 1000 cells resulting in stochastic problems. However, the number of cells inserted to RNA isolation reached from minimal 40.000 up to 1.000.000 cells in this study. In the first step of WTA reaction, a mixture of random and oligo-dT primers were used for cDNA generation. Based on the primers, it is recommended to analyze no full-length transcripts after WTA. In this study no full-length transcripts were analyzed by RT- and qPCR and the following example revealed that the length of PCR products could also not be the explanation for undetectable *HOX* transcripts after WTA reaction. The product length of *GAPDH* is 238 bp. In qPCR analysis, *GAPDH* was stable detected in 9 out of 9 tested WTA samples. The PCR product length of *HOXD3* is 187 bp. Although the PCR product length of *HOXD3* is smaller compared to *GAPDH*, *HOXD3* was only detected in 3 out of 9 WTA samples by qPCR. For SYBR Green assays, the amplicon for PCR products should be less than 300 bp [3]. This aspect was also taken into account for qPCR analysis since the PCR products exhibit a length between 112–238 bp. In summary, neither the RNA quality nor the quantity of RNA template for WTA reaction, the amount of cDNA template for PCR reaction, the number of cells for RNA isolation or the length of PCR products seemed to be responsible for the undetectable low-copy *HOX* transcripts in RT- and *q*PCR analysis after WTA.

The undetectable low-copy *HOX* transcripts may be a result of unspecific amplification during WTA reaction, perhaps of random extension of primer-dimers. In case of unspecific amplification, at first the low-copy transcripts get lost, then the number of medium-copy transcripts is impacted, while the influence on high-copy transcripts is unaffected. However, the customer has no influence on this effect, provided that RNA degradation and contamination can be excluded. Additionally it cannot be excluded that the different primers used for normal cDNA synthesis by standard reverse transcription (oligo-dT primers) and for cDNA synthesis by WTA (mixture of random and oligo-dT primers) contribute to the significantly reduced detection of *HOX* transcripts after WTA compared to non-amplified BM-MSC samples. The mixture of random and oligo-dT primers was probably used for cDNA synthesis by WTA to also recognize transcripts without poly(A) tail. Therefore, it is contradictory that less *HOX* transcripts could be detected after WTA compared to standard reverse transcription using only oligo-dT primers for cDNA synthesis.

In the second step of WTA reaction, the generated cDNA is ligated before amplification. The ligation is required because the amplification enzyme *phi29* DNA polymerase only recognizes cDNA fragments longer than 2 kb [4]. Another research group modified the ligation and amplification time of WTA reaction and identified the ligation step as key factor [19]. Their experiments revealed a correlation between the ligation time and the amplification efficiency. Higher amplification efficiencies were obtained with increasing ligation time. The efficiency of ligation may also be an explanation for significantly reduced detection of *HOX* transcripts after WTA in line with their expression level.

The generation of cDNA in the first step of WTA reaction, mainly led to cDNA fragments smaller than 2 kb. If these cDNA fragments are not ligated during the ligation step of WTA reaction, they cannot be amplified by *phi29* DNA polymerase afterwards. Therefore, the representation of original transcriptome may depend on both, the amplification and ligation efficiency of WTA reaction.

In the study presented here, the WTA efficiency was not equal for medium- and low copy *HOX* transcripts of human BM-MSCs. Moreover, the low-copy *HOX* transcripts were not fully converted into cDNA by WTA reaction resulting in false negative RT- and qPCR data. The represented qPCR data of human BM-MSCs can lead to the conclusion that the WTA efficiency may depend on the expression level of the respective gene. Furthermore, it can be suggested that this is not a specific effect of *HOX* transcripts and most likely also applies to other genes tested in other cell types. The use of RNA amplification is becoming more popular, as often only limited amounts of cells are available for gene expression analysis, but representing the complete original transcriptome, especially with regard to low-copy transcripts, is error-prone.

## Supporting Information

S1 Table Primer sequences. (DOCX)
