# Supplementary material for: RNA Amplification Protocol Leads to Biased Polymerase Chain Reaction Results Especially for Low-Copy Transcripts of Human Bone Marrow-Derived Stromal Cells
Source: PLoS One. 2015 Oct 20;10(10):e0141070. doi: 10.1371/journal.pone.0141070 (PMC4613830; doi:10.1371/journal.pone.0141070)
Supplement: S1 Table — (DOCX) [file pone.0141070.s001.docx]

| **Gene** | **Sequence 5’→ 3’** | **Product length** |
| --- | --- | --- |
| ***HOXA1*** | for: TCGGAGACATCTTCTCCA, rev: CAGGTACTTGTTGAAGTGG | **178 bp** |
| ***HOXA2*** | for: TCAGCCACAAAGAATCCCT, rev: CTCTCAGTCAAATCCAGCA | **175 bp** |
| ***HOXA3*** | for: TCAGAATGCCAGCAACAAC, rev: AGTGAGGTTCAGCAGATTG | **310 bp** |
| ***HOXA4*** | for: TGGATGAAGAAGATCCATG, rev: TGGTCTTTCTTCCACTTCA | **233 bp** |
| ***HOXA5*** | for: TAAGTCATGACAACATAGGC, rev: TTAAACGCTCAGATACTCAG | **276 bp** |
| ***HOXA6*** | for: ACTACCTGCACTTTTCTCC, rev: CGTGGAATTGATGAGCTTG | **359 bp** |
| ***HOXA7*** | for: TCCTACGACCAAAACATCC, rev: GTCCTTATGCTCTTTCTTCC | **324 bp** |
| ***HOXA9*** | for: AATGCTGAGAATGAGAGCGG, rev: TCTCGGTGAGGTTGAGCAG | **208 bp** |
| ***HOXA10*** | for: GATTCCCTGGGCAATTCC, rev: ACTTGTCTGTCCGTGAGG | **191 bp** |
| ***HOXA11*** | for: AACTTCAAGTTCGGACAGC, rev: AGACGCTGAAGAAGAACTC | **230 bp** |
| ***HOXA13*** | for: TACCTGGATATGCCAGTG, rev: GTATTCCCGTTCAAGTTC | **279 bp** |
| ***HOXB1*** | for: CAAGACAGCGAAGGTGTCA, rev: CTTCTGCTTCATTCGTCGG | **208 bp** |
| ***HOXB2*** | for: TCCACCCTTCAGAGACCCA, rev: GCGCGTGACAGCAGGCTT | **271 bp** |
| ***HOXB3*** | for: CAAATCTCCTTGGACCGGCTGTTG, rev: GTTCCAAGCGGCTGACCTTAG | **282 bp** |
| ***HOXB4*** | for: GCAAAGTTCACGTGAGCA, rev: TTGGGCAACTTGTGGTCT | **238 bp** |
| ***HOXB5*** | for: ATCAGCCATGATATGACCG, rev: GTTGTCCTTCTTCCACTTC | **207 bp** |
| ***HOXB6*** | for: GAATTCGTGCAACAGTTCC, rev: TATCTTGATCTGCCTCTCC | **175 bp** |
| ***HOXB7*** | for: GAGTAACTTCCGGATCTACC, rev: TGATCTGTCTTTCCGTGAGG | **182 bp** |
| ***HOXB8*** | for: TACGCAGACTGCAAGCTTG, rev: TTTGCTGCTGGGGAACTTG | **324 bp** |
| ***HOXB9*** | for: TGCTGTCTAATCAAAGACC, rev: AGAAACTCCTTCTCTAGCT | **175 bp** |
| ***HOXB13*** | for: AGCATTTGCAGACTCCAGC, rev: TGTTCTTCACCTTGGCGAG | **251 bp** |
| ***HOXC4*** | for: AATTCACGTTAGCACGGTG, rev: AGTGGTCTTCAGAAGTACC | **317 bp** |
| ***HOXC5*** | for: TGACCAAACTGCACATGAG, rev: TTCTTCCACTTCATCCTGC | **205 bp** |
| ***HOXC6*** | for: ATGCAGCGAATGAATTCGC, rev: GTGGATGTGAGATTAGATTC | **239 bp** |
| ***HOXC8*** | for: CCAACACTAACAGTAGCGA, rev: GATCTTCACTTGTCTCTCG | **233 bp** |
| ***HOXC9*** | for: AAGCACAAAGAGGAGAAGG, rev: GTTTAGGACTGCTCCTTGT | **281 bp** |
| ***HOXC10*** | for: AGACACCTCGGATAACGAAG, rev: AATGGTCTTGCTAATCTCCAG | **190 bp** |
| ***HOXC11*** | for: TTTCTTCGACAACGCCTAC, rev: TCCGTCAGGTTCAGCATC | **360 bp** |
| ***HOXC12*** | for: AATCCGACTCCAGTTCGTC, rev: TCTGCCAGTTGCAACTTCG | **184 bp** |
| ***HOXC13*** | for: TGTACTGCTCCAAGGAGCA, rev: CTTCTCTAGCTCCTTCAGC | **152 bp** |
| ***HOXD1*** | for: TCTAAGAAAGGCAAACTCGC, rev: GTGTCATTCAGGTGCAAGC | **170 bp** |
| ***HOXD3*** | for: AGCAGAAGAACAGCTGTGC, rev: GTGAGATTCAGCAGGTTGG | **187 bp** |
| ***HOXD4*** | for: ATGAAGAAGGTGCACGTGA, rev: TGTGAGCGATTTCAATCCG | **160 bp** |
| ***HOXD8*** | for: TGAGACCACAAGCAGCTCC, rev: GTCTTCCTCCAGCTCTTGG | **112 bp** |
| ***HOXD9*** | for: CAACTTGACCCAAACAACC, rev: ACCTGTCTCTCTGTTAGGT | **182 bp** |
| ***HOXD10*** | for: CAAGAGTACAATAATAGCCC, rev: GGTGTATCAGACTTGATTTC | **278 bp** |
| ***HOXD11*** | for: AAGAGCAGCAGCGCAGTTGC, rev: AGGTTGAGCATCCGAGAGAG | **149 bp** |
| ***HOXD12*** | for: AACTTGAACATGACAGTGC, rev: TATTGGACAATTCCTTGCG | **202 bp** |
| ***HOXD13*** | for: ATATCGACATGGTGTCCAC, rev: CCGCTTGTCCTTGTTAATG | **302 bp** |
| ***GAPDH*** | for: GAGTCAACGGATTTGGTCGT, rev: TTGATTTTGGAGGGATCTCG | **238 bp** |
| ***RPL13A*** | for: GAGGTATGCTGCCCCACAAA, rev: TTCAGACGCACGACCTTGAG | **136 bp** |
